# Opportunities to inform German residents about the possibility of skin cancer screening and to inform stakeholders to take appropriate actions: A qualitative approach

**DOI:** 10.1002/cam4.5717

**Published:** 2023-04-16

**Authors:** Theresa Steeb, Anja Wessely, Markus V. Heppt, Michael Erdmann, Stefanie J. Klug, Carola Berking

**Affiliations:** ^1^ Department of Dermatology University Hospital Erlangen, Friedrich‐Alexander Universität Erlangen‐Nürnberg Erlangen Germany; ^2^ Comprehensive Cancer Center Erlangen—European Metropolitan Region of Nürnberg Erlangen Germany; ^3^ Epidemiology, Department of Sport and Health Sciences Technical University of Munich Munich Germany

**Keywords:** health care utilization, health promotion, interviews, mass screening, melanoma, participation rate, qualitative research, secondary prevention, skin cancer, skin cancer screening

## Abstract

**Background:**

The national skin cancer screening (SCS) was introduced in Germany in 2008. However, public awareness and participation rates remain low. There are no campaigns or target group‐specific invitation strategies for SCS yet. Thus, our aim was to derive potential suggestions on how to best inform German residents about the possibility of SCS.

**Methods:**

Semi‐structured, individual interviews with male and female German residents aged ≥35 years were conducted in Erlangen (Germany) to explore opportunities on raising awareness of SCS. Interviews were audiotaped, transcribed verbatim, and analyzed using qualitative content analysis.

**Results:**

Overall, 39 persons were interviewed. About 79.5% (31/39) had already undergone at least one SCS. Numerous opportunities to raise awareness of the possibility of SCS were suggested which were categorized into three main topics: the role of public promotion, health‐related caregivers, and health insurance. Similar themes were identified for inviting entitled persons to undergo SCS after 2 years. Furthermore, age‐dependent communication approaches were proposed, that is, younger persons should be approached electronically, while the older generation should be targeted with traditional media like mail.

**Conclusions:**

The results of this project will inform stakeholders to take appropriate actions. The findings may contribute to increase participation rates in SCS and thus lead to earlier detection of skin cancer.

## 1 INTRODUCTION

Skin cancer is the most frequent malignant neoplasm in light‐skinned individuals.^1^, ^2^ The incidence of melanoma and keratinocyte cancer has steadily increased in recent years and the trend is still ongoing.^1^, ^3^ The tumor stage at the time of diagnosis influences the survival rate although promising new therapy strategies for advanced disease have been developed in recent years.^4^, ^5^ Screening for skin cancer as a form of secondary prevention has the potential to reduce mortality and morbidity by detecting tumors at an earlier stage with a better prognosis.^4^, ^6^ Following the pilot project “Skin Cancer Research to provide Evidence for Effectiveness in Northern Germany” (SCREEN), the unique national population‐based skin cancer screening (SCS) was implemented in Germany in 2008.^7^, ^8^ Importantly, SCS programs like a community‐based screening program in Queensland, Australia,^9^ the screening program at the Lawrence Livermore National Laboratory (LLNL) in California, USA^10^ have already been attempted in other countries. However, the German SCS program is unique as it is the only nationwide program for the early detection of skin cancer that has been successfully implemented.^11^ It involves a voluntary, standardized full‐body examination by dermatologists and general practitioners (GP). The costs for this examination are reimbursed by the statutory health insurance funds every 2 years for all members over 35 years of age.^12^

Since the introduction of SCS in Germany, there has been an increase in the incidence of skin cancer, especially in the early and prognostically more favorable stages.^13^ Furthermore, a decrease in age‐standardized melanoma mortality has been observed.^14^, ^15^ However, the participation rate is restrained: only about one in two eligible persons has taken advantage of the screening so far.^16^ Other estimates assume that participation rates in SCS range between 24% and 39%.^12^, ^16^, ^17^, ^18^ In contrast to the invitation programs of other cancer entities, no campaigns or target group‐specific invitation strategies currently exist to enhance the participation rates of SCS. The current state of research shows that invitation strategies for SCS have been understudied. Little is known about how participation rates might be increased and no qualitative research has investigated this topic before. Thus, we were interested in how we can raise awareness for SCS. A qualitative approach was used to investigate this aspect. Qualitative approaches, including individual interviews, give voice to those whose views are rarely heard.^19^ Besides, they enable the performance of initial research to develop theories and hypothesize and even test them; and to seek explanations.^19^ Here, we report the results of explorative, individual qualitative interviews in order to derive potential suggestions for stakeholders on how to best inform German residents about the possibility of SCS. Our findings will contribute to increase SCS participation rates.

## 2 MATERIALS AND METHODS

The HELIOS project (German acronym for: skin cancer‐specific invitation suggestions to participate in the SCS program) consists of^1^ semi‐structured explorative qualitative interviews followed by^2^ a quantitative online survey. The protocol for the entire project has been published previously.^20^ This manuscript reports on a qualitative investigation from the first part of the project on opportunities to inform and remind the general population about the possibility of SCS. To date, no regular reminders are sent to people that can participate in the SCS program.

### 2.1 Setting and participants

Individual, face‐to‐face interviews were conducted in German and audiotaped (Olympus‐ LS‐P1) between February and August 2021 at the Department of Dermatology at the University Hospital Erlangen by the first author (TS, postdoctoral researcher). The study included a convenience sample of male and female participants ≥35 years. Hence, age was the only inclusion criterion. The participants were recruited by flyers in the University Hospital as well as advertisements on the intranet and the homepage of the University Hospital Erlangen. Thus, medical staff as well as patients could participate.

### 2.2 Data collection

Prior to the individual interviews, a semi‐structured interview guide was developed by the investigators.^21^, ^22^, ^23^ It included open‐ended questions covering the participants' previous experience with SCS, their interest in SCS invitation procedures, preferred communication strategy, and the evaluation of the information brochure “Detecting skin cancer” of the German Cancer Aid to obtain suggestions for improvement.^24^ Follow‐up and probing questions were used for clarification and elaboration. Two pretest interviews were performed a priori to pilot the semi‐structured guide, which were included in the analysis. Besides this, a questionnaire was dispensed to obtain socio‐demographic data and to ask participants whether they had already been affected by skin cancer. The full semi‐structured interview guide is available in the supplementary material of the published protocol.^20^

### 2.3 Consent

All participants provided written informed consent to participate in the qualitative interviews. All participants were identified by pseudonyms to ensure anonymity. They received an incentive of €40 as reimbursement for their time. The HELIOS study was approved by the Ethics Committee of the Medical Faculty at the University Hospital Erlangen, Germany (Vote number: 188_20B).

### 2.4 Data analysis

Recorded interviews were transcribed verbatim by TS, AM, and NG and a thematic analysis was performed using the computer‐aided software MAXQDA (VERBI Software—Consult‐Sozialforschung GmbH).^25^, ^26^ Selected quotes were translated by a native speaker. Socio‐demographic data were described descriptively.

## 3 RESULTS

### 3.1 Characteristics of the sample

Overall, 26 women and 13 men with a median age of 47 years (range 35–79) participated in the interviews (Table 1). Eight of them had been diagnosed with skin cancer (melanoma: 5, basal cell carcinoma: 2, basal cell carcinoma + actinic keratosis: 1). In total, 79.5% (31/39) had already at least one SCS. Nearly all had statutory health insurance (89.7%, 35/39). Furthermore, 14 participants were married and lived together, 14 were single, six stated to be divorced, two to be married but not to live together, and another two answered to be widowed. Interviews lasted between 11:32 and 60:24 min (mean 26:24 min). No differences were identified between the baseline characteristics of participants with skin cancer and those without.

**TABLE 1:** Overview of the characteristics of the 39 participants.

Gender	
Male	13 (33.3%)
Female	26 (66.7%)
Skin cancer (Year of diagnosis (range): 2012–2020)	
Melanoma	5
Basal cell carcinoma	3
Actinic keratoses	1
No prevalent skin cancer	31 (79.5%)
Previous participation in skin cancer screening	
Yes	31 (79.5%)
No	8 (20.5%)
Health insurance	
Statutory health insurance	35 (89.7%)
Private health insurance	4 (10.3%)
Family status	
Married	16 (41.0%)
Single	14 (35.9%)
Divorced	6 (15.4%)
Widowed	2 (5.1%)
Education	
Higher education entrance qualification/Abitur	18 (46.2%)
Secondary school certificate	13 (33.3%)
High school diploma	1 (2.6%)
Technical college certificate	4 (10.3%)
No school certificate	1 (2.6%)
Other	1 (2.6%)
Missing	1 (2.6%)
Employment	
Full‐time	26 (66.7%)
Part‐time	6 (15.4%)
Not employed	5 (12.8%)
Residence	
City	13 (33.3%)
City outskirts	15 (38.5%)
Rural	11 (28.2%)

### 3.2 Initial awareness of SCS

First, we were interested in how the participants initially became aware of the SCS program. Six participants were informed about SCS by their treating GP. One GP informed them as part of the Check‐up 35, another health prevention program. Many participants had heard of SCS by friends (*n* = 2) or family (*n* = 3) or as part of their education in a health‐related field (*n* = 5). Three participants had received information material from their health insurance, and two via the bonus program of their health insurance while another two became aware of SCS while searching for services their insurance offers.

Two participants also stated to have become aware of SCS by flyers available in waiting rooms. Additionally, other participants stated to have heard of SCS for the first time at their workplace, by the press, on their own initiative, by an alternative practitioner, or since they have participated in a recruiting study which recommended SCS.

### 3.3 Opportunities to raise general awareness about SCS

When asked how the general awareness of the possibility of SCS could be increased, three themes were revealed: the role of public promotion, health‐related caregivers, and health insurances. Selected quotes are presented in Table 2. Overall, the importance of public promotion was emphasized the most, for example, via social media (*n* = 7) such as Instagram™ (*n* = 2) or Facebook™ (*n* = 1). Traditional media (TV [*n* = 6], radio [*n* = 5], teletext [*n* = 1], billboard [*n* = 7], flyers [*n* = 4], or advertisements in magazines [*n* = 1]) were mentioned as well. Participants also encouraged the information distribution by mail, with or without a fixed appointment for SCS. Notably, some participants proposed to use social media and apps for the younger target group while the older target group should be approached via other opportunities: “So I think it would be good, similar to mammography, if information is sent to patients with a certain age (…). And then adapted to the age of the patient, the younger generation, of course, more via social media and the older generation, of course, more via the health insurance company or via a family doctor” (P30).

**TABLE 2:** Common themes and selected quotes raised by the 39 participants to increase awareness for the opportunity to undergo skin cancer screening.

Themes	Sub‐category and quotes
Public promotion	New and social media (e.g., Instagram™ or Facebook™) “Such an app would also be quite good. But maybe only for a certain age group, because people over 60 might have difficulties with the app, as they do with the Corona app now. So I have a toothbrush, an electric one, that reminds me to buy a new brush head after 3 months.” (P34) “or the other media, what we have there, Instagram, Facebook” (P34).
	Traditional media for advertisement (e.g., TV, radio, teletext, billboard, flyers or advertisements in magazines) “that this really happens when you just go to work and walk past such a poster, where the key points are really there now, let us advise you if you want. That it simply comes into consciousness without actually going into the individual's mailbox, but that it becomes visible, visible. Yes. I think posters are good (…) In the meantime, there are also TVs in buses or trains with information, where news is shown or something like that. If you put ads on them (…) or there are always ads in front of them on YouTube. You could use them for something good.” (P39) “I do not think it's bad at all, what I do not think is bad at all is, for example, posters or small ones like that at the bus stop or something like that or in underground stations, there are also messages like that, and if you give that, I say, a serious face and it's not too lurid but simply health information and an appeal, I think that's quite useful to reach the masses.” (P32)
	Personal communication (mail, with/without a fixed SCS appointment, short text message) “So I would find it very good if you were informed every 2 years. With colonoscopies, you are already informed that maybe the 5 years are up again, and with mammograms, this programme is in place until you are 70, so that you get an invitation by mail every year.” (P6) “At my dentist, they just send me a text message. The typical one or an email, which is what many doctors' offices do anyway for appointment reminders, hairdressers already do.” (P34) “And I think to myself, if it has a different format, now last week I got a reminder from the TÜV [=German company that tests and certifies technical systems and objects as e.g., cars], I thought it was really cool, it was just a card, it was so big, it said my number plate and TÜV May 2021, that's such a card, I quickly hang it on my notice board or have it somewhere, then I know, ah, I still have to do it.” (P7) “Yes, I think there is definitely also SMS notification, WhatsApp notification, by letter. For example, I always get a reminder from the dentist every six months that the dental check‐up is due, and the dentist offered that once, and that's why I was happy to agree that he could save the data and remind me, because it really used to be that you simply forget that six months or a year has passed. And it's probably the same with skin cancer screening, no matter which examinations. You always think it was yesterday and then when you look it up it was, or it was half a year ago and then when you look it up it's actually been longer. That's why I think that reminders are definitely useful and yes, by SMS, by WhatsApp by (…) yes, by email everything, everything that somehow reminds you personally.” (P20)
	Testimonials (affected persons, famous persons) “In the case of bowel cancer, people have even been found who are in the public eye, actors who advertise for it, but I have not seen anything in the media about skin cancer yet.” (P8) “Maybe celebrities or something, I mean, they have tried that with Corona or something. There are probably also people who once had skin cancer or were affected or something, and if you somehow present individual fates to people, that hits a nerve rather than saying that skin cancer is a topic or something, something like that, you find it personalized and connected with the face” (P10)
	Other (campaign days/week, family/friends) “Perhaps this could also be offered somehow in such an action week, simply the vacant ones, when the corona vaccination centres, I say, are dismantled, one could make a skin cancer screening centre there, where one actually offers a skin cancer screening to all interested people once or something, or draws a number or has an appointment made.” (P32) “Most people only become sensitive to it when they suddenly hear that someone in their acquaintance or somewhere in the village or in the immediate vicinity is dying of a melanoma or skin cancer, who was perhaps relatively young.” (P9)
Health‐related caregivers	Medical disciplines (gynecologists, urologist, pediatricians, dentists): either actively screening or informing “Or that the doctors then explicitly point out again during the examination that the possibility exists (…) So maybe the subject area does not fit here, but I definitely go to the dentist once a year (…). So I think I would be reached the most if I was somehow directly pushed again by a doctor or something.” (P38) “Maybe more through company doctors, where you are of course obliged to go regularly. So I think you are more likely to reach someone there than with doctors in private practice. They do not have to do it, but they have to point out, ok, the last time you came to us was 3 years ago, what kind of preventive medical check‐up did you have, that might be good to do there, maybe something like that, yes. To do it through the company doctors, because I think no matter where you are employed, there is a company doctor almost everywhere.” (P30) “So the family doctor is important. I think it's a decision‐making thing, well (…) the younger people often do not have a family doctor, I mean later you have a family doctor, but if you want to reach the younger ones, how do you reach them, that's the gynecologists, for women it's the gynecologists, from 20 onward there's breast cancer screening. Another idea would be to reach them through specialists.” (P24)
	Role of general practitioners “At the family doctor's, yes, I'm there with a cold, flu, well, I do not feel well there, but I'm there for something else, I just need something, and he can say, look out, let us do the skin screening as well.” (P8) To be honest, I would also find it very good if the general practitioner, when asked about it, sees that the patient is already at an age where this is due. That you really address them directly.” (P37) “And I think older people, if they do not have something wrong with their skin, sometimes they do not even notice that they have something wrong with their skin. That's why it's important that the family doctor looks at it and says what your skin actually looks like and then refers you on.” (P34) “I think the best way is actually through the family doctor. Everybody goes to the family doctor at least once a year, I would say, and they should perhaps give their patients a nudge every now and then, a hint as to whether they would like to have another skin screening or whether they have been to the dermatologist again. I think that makes the most sense and is the most cost‐effective.” (P36) “The reminder from the doctor is a bit more binding because it comes from a human being, even if it is certainly printed by a computer. But there is a message from my doctor and if I do not go, then maybe he asks again four weeks later and says, “Have you seen it, do you really not want it or have you just forgotten?” It would just be important.” (P5)
	Waiting area of doctor's offices “I was at my general practitioner's last week and saw the sign in the waiting room “Check‐Ups including skin screening here” and of course that was the opportunity.” (P28)
	Health‐related disciplines/lifestyle (gym instructors, hairdresser, physiotherapists, pharmacists) “Physiotherapists do not always see the patient fully dressed, but when they give massages or do physiotherapy, certain parts of the body are usually not clothed, be it the upper arms or the feet or the face. So I can imagine that this would be a contact person (…). But I would also welcome beauty salons, because they can see the skin at least up to the breast area, hairdressers, but I do not know how far (…) screening is in the hair.” (P6) “The hairdressers do, even mine always notices when combing, oh you have a birthmark there. It would be good to at least inform them that if there is something, just tell the client, that would be an idea.” (P26) “Whereas you can actually also find it in rehabilitation facilities or simply in health‐related facilities, such as health training, nutrition (…). Simply where people go who otherwise have an increased interest in health topics, that you really come across the information directly.” (P39) “If it goes to the elderly in care, then it would also be organizations that take care of them. Something like that in nursing homes might be another idea.” (P26)
Health insurances	Advertising SCS via mail or social media or apps “Or that maybe even the health insurance company writes to you when you reach a certain age, that then really every health insurance company simply takes the initiative and says now you have turned 35, now you have the opportunity to participate.” (P33) “So I would say that at least I would personally estimate that currently everyone up to 70 has a smartphone and either the reminder comes simply via the health insurance app. So everyone I know personally usually has their health insurance company as an app on it and yes, because of bonus programmes or because of information or because it simply costs nothing in principle. Whether it's the 87th app with some games or the 87th health care app, then the health insurance is also OK in my case. And I think that a reminder via such a function makes sense, that you can, so as I said, I can enter it in my bonus. I have to enter the date, then in principle the function of the app would be relatively simple, simply to say, hey in three quarters of a year, how does it look […] Time's up again, they could go back to screening. You entered this two years ago, would you like to make an appointment. Somehow something like that I think is relatively good to remember.” (P26) “I would not install an app just to be notified of the skin cancer screening, but if it was done via the health insurance app, that would be fine with me, absolutely, it's like push notifications. Exactly. So I would have to use the app, I would not install an app specifically for that. Yes. So if it's somehow done via the insurance company, or I'll say in the future, telematics, infrastructure, if there are any apps, that would be ideal, of course.” (P38) “In fact, I would have been pleased to receive a flyer in the post, perhaps even from the health insurance fund directly by email or mail, there is a new one now, you can have it done. I think in the case of cervical [cancer] screening, the health insurance fund had sent very specific information by mail and that would actually have been there, I would have been pleased too.” (P39)
	Incentives via persisting bonus programmes “There used to be (…) a bonus programme (…) that said if you did the SCS at regular intervals, you would get a bonus back (…).” (P34) “Many health insurance companies have a preventive care bonus programme where you can collect points if you have had all your preventive examinations in various disciplines and can somehow show that, or they actually see it, they actually have it in their data, that you can somehow, I do not know, acquire any discount campaigns or special offers or something in a bonus programme. I think many people would jump at it. “(P31)
	Bonus‐penalty system “I would link it, quite simply: (…) away from this complete voluntariness, but toward a separate voluntariness. In other words, there are two solutions: either he gets a reward for going to his check‐ups, or he gets a penalty, which means his contribution to the fund will be higher. Either one way or the other. “(P33)

Another five participants mentioned that testimonials should advertise SCS and inform about it. Two participants explicitly stated that skin cancer patients should promote SCS while the other participants suggested famous or well‐known persons to promote SCS: “(…) if you somehow present individual fates to people, that hits a nerve rather than saying that skin cancer is a topic (…), you find it personalized and connected with the face” (P10). Interestingly, participants who had never undergone SCS before suggested more often social media and public promotion like TV/radio.

Sixteen participants suggested that other medical disciplines like gynecologists, urologists, pediatricians, or dentists should be involved in performing SCS or informing about the possibility. Nine participants stated that GP could be more included in raising awareness by directly informing and addressing their patients as they have a trusting bond. Other possibilities included the distribution of brochures in the waiting room of doctor's offices (*n* = 2). Furthermore, employers (*n* = 2) or company doctors (*n* = 5) should inform their employees about SCS. The participants especially emphasized their role in approaching outdoor workers. Other disciplines should also be encouraged to actively approach people and promote SCS, such as gym instructors, hairdressers, or pharmacists.

Notably, the importance of the health insurance in either advertising SCS via mail or social media (*n* = 8) or by creating incentives via persisting bonus programs (*n* = 4) was highlighted: “Or that maybe even the health insurance company writes to you when you reach a certain age, that then really every health insurance company simply takes the initiative and says “now you have turned 35, now you have the opportunity to participate” (P33). Furthermore, one participant suggested that a bonus‐penalty system should be introduced. Besides, information on SCS and other secondary prevention programs should also be incorporated into the school curriculum, stated six participants.

Establishing a recurring campaign day/week, mobile screenings, launching screening centers similar to the COVID‐19 vaccination centers, compulsory distribution of information when booking a holiday, having travel consultation or when using tanning beds were also mentioned. Lastly, the participants also stated that relatives and friends should raise awareness (*n* = 2) and that general awareness opportunities should be made available in various languages (*n* = 1).

### 3.4 Opportunities to remind on SCS

Since regular participation is important for the early detection of skin cancer, we also asked the participants for reminder opportunities. In general, similar themes were revealed as for raising general awareness. Many of the interviewees stated that health insurance should approach eligible members (*n* = 5). Two of those stated that the insured members should indicate their preferred reminding procedure a priori. Eighteen participants stated that a reminder by mail should be sent, while seven wished for a fixed appointment. One of them also suggested that mail in combination with a fixed appointment should be realized for all available screenings. Furthermore, nine participants suggested sending reminders by email, however, some were also against it as they would consider such emails to be spam. Furthermore, some recommended a reminder note via text messaging, such as WhatsApp or a short message service (*n* = 6). A reminder app for SCS was suggested by 11 participants, however, another seven emphasized that they do not see a need for an SCS‐specific app but rather for a reminder app integrating all screening examinations. Importantly, some of the participants were also skeptical against an app: “If (…) my mobile phone breaks down and I need a new one and not everything is so far advanced with the installation. Then the memory is gone again, and I say to myself, OK, I've got my card or my letter on it, or my emails, and I don't delete them straight away. For me, that's better than an app like this. Or an app can also have a problem, an error that has to be fixed first (…) so I'm still a bit skeptical” (P35) and “I somehow have the feeling that I have so many apps and now I have the Corona app and the vaccination app and now the catastrophe app, so I don't want to have another app.” (P24).

Four respondents also proposed a phone call as a reminder. In addition to that, seven interviews recommended that reminders should depend on the age, that is, younger persons should be approached electronically, while the older generation should be targeted with traditional media like mail. Other suggestions included a reward in general for regular participation (*n* = 3) or a bolus‐penalty‐system.

Besides, seven interviewees also highlighted that the GP should remind them during consultations. Furthermore, three suggested a compulsory follow‐up appointment and that is for example, the doctor's receptionist should ask them for the next appointment.

### 3.5 Wishes for SCS

When asked about wishes regarding SCS, the majority revealed that the topics of SCS, skin cancer, primary and secondary prevention should be addressed in school (*n* = 6) and kindergarten (*n* = 1). Especially participants who had already been affected by skin cancer emphasized this. Three participants wish for more usage of artificial intelligence in SCS or that photos are taken routinely for comparison (*n* = 1). Besides, two participants criticized the screening interval of 2 years: “My opinion is that you go to the gynecologist and dentist once a year. If you had a rhythm like that, not every two years, but once a year, people would remember that.” (P29).

Additionally, the participants desired that the physician would spend more time on SCS (*n* = 1), to receive a clear assessment after SCS (*n* = 1), improved management of future appointments (*n* = 1), that the SCS program is also advertised in other languages (*n* = 1) and with a stronger focus for participants below the age of 35 years (*n* = 1). Furthermore, one participant appealed to rename SCS as it may deter potential participants: “(…) the word itself, in my opinion, is very badly chosen, that it says skin CANCER and the word cancer, if it appears somewhere, is always negative for everyone, and that's why people have respect for it (…) Dental prophylaxis, yes, (…) for example (…) is a positive word.” (P24).

### 3.6 Evaluation of the information brochure

Seven of the 39 participants said they like the information brochure “Detecting skin cancer” of the German Cancer Aid^24^ in its current form and would not modify anything, but ten criticized the brochure as too long and five as too difficult to read or understand. Four participants suggested putting a short summary of the most important information on the first page of the leaflet and highlighting important information in the entire brochure (*n* = 5). Another four proposed to include contact details (e.g., as a sticker on the back of the leaflet) of GP and dermatologists that perform SCS. “where I can look for doctors carrying out this skin cancer screening. That it [the leaflet] might refer to it with a QR code or something similar” (P7). Including more pictures of different types of nevi or the examination procedure were suggested by five participants: “Not the blatant pictures of some kind of cancer, of course. It should not scare people, but it should be about examinations or something like that, and maybe also about the equipment used for the examination, because nobody can imagine anything” (P37). Interestingly, three participants wished for more frightening pictures, while another one explicitly discouraged the use of such photos and would rather deploy positive examples. Further suggestions included more information regarding primary prevention (*n* = 1) and translations of this leaflet into other relevant languages (*n* = 1).

## 4 DISCUSSION

SCS is considered to be a useful measure that is not very costly for the patient and approved by a large part of the population.^12^ Nevertheless, it remains controversial and its benefits, risks and relevance have heatedly been discussed.^27^, ^28^ Most importantly, the effectiveness of SCS widely discussed among specialists. Some studies argue that SCS fails to decrease skin cancer mortality rates but may increase the risk for overdiagnosis.^29^, ^30^ However, other studies from Germany showed that SCS reduces melanoma mortality.^7^, ^31^ More than 10 years after its introduction, participation rates have increased from 38% in 2013 to 45% in 2015, but still less than half of those entitled have ever taken advantage of this preventive measure.^16^, ^32^ Surprisingly, nearly 80% of our sample had already undergone at least one SCS which might be due to the sampling strategy as we recruited in a hospital‐based setting. These participation rates are much higher and thus contrary to previous studies where for example only about one in two eligible persons has taken advantage of the screening so far.^16^

Interestingly, recent studies have shown that non‐participation is mostly not based on an informed decision against screening, but more often on ignorance of the offer or misconceptions about the principle of screening.^12^, ^16^ Previous research has shown that only one out of two study participants knew about the SCS program; the effect was more pronounced among women and those aged more than 35 years.^16^ Thus, a risk group‐oriented education of the population about the relevance of skin cancer is indicated. In particular, the male and middle‐aged groups must be better reached. More than half of the people who have not yet undergone SCS stated to feel healthy.^33^ Additionally, a connection between participation in screening and socio‐economic status has been identified: The lower the level of education, the lower the probability of participation in screening.^12^, ^16^, ^33^ Furthermore, many had not participated due to other prioritized diseases or lack of time.^12^, ^33^ Hence, there is an information deficit and a lack of risk awareness about skin cancer.^33^, ^34^, ^35^ Nevertheless, most people appreciate the possibility to get a SCS and, additionally, informed persons use the offer of SCS more frequently than uninformed persons.^16^

We were interested in how we can raise awareness for SCS among eligible persons. In our sample, the three main themes of public promotion, health‐related caregivers, and health insurances were identified. Many of the participants stated to have been informed about SCS by their GP, through information distributed by the health insurances, public media promotion, or relatives or friends. This is in line with a nationwide cross‐sectional study which revealed that most had been told about SCS by their GP, followed by their health insurance, dermatologist, via media such as TV, radio, the internet or newspapers, or through relatives or friends.^32^ In the interviews, the responsibility of health insurances in approaching their members was mentioned. Health insurances have direct access to their clients for health education and distributing information. Although information opportunities and campaigns already exist, such as bonus programs for preventive examinations, these have been largely discontinued. Thus, the educational work by health insurance managers is expandable and should be approached more proactively.^33^

Overall, 39 participants were recruited for the interviews. This sample size is still rather small and the qualitative approach limits the generalizability of our findings. Besides this, sampling bias cannot be neglected as the participants were recruited via flyers in the University Hospital as well as advertisements on the intranet and the homepage of the University Hospital Erlangen. Another limitation is that mainly female participants >35 years participated in the interviews, who are often better informed about cancer screening programs.^18^ Thus, objectivity of results such as the high participation rate might be questionable. Additionally, no people with skin of color, recent migrants, and only a few people with a low socioeconomic status were willing to participate. Thus, we cannot rule out the possibility that we missed important opinions and suggestions from these groups.

Interestingly, smartphone apps were mentioned as another opportunity for raising awareness on SCS or reminding, preferably for all secondary prevention examinations. This might be explained by the fact that the participants in our sample were rather young with a median age of 47 years (range 35–79) and are thus familiar with the use of smartphones and apps. A previous systematic search on skin cancer‐related apps available in German identified apps that featured the tracking or mapping of moles, risk calculators, general information on prevention or education, or timers for sunscreen application.^36^ However, some interviewees were also skeptical about new technology. In another study, even those already suffering from skin cancer showed little interest in skin cancer‐related apps.^37^ However, further research suggests that melanoma patients and unaffected individuals are open to artificial intelligence in skin cancer diagnostics.^38^

## 5 CONCLUSIONS

**FIGURE 1:**
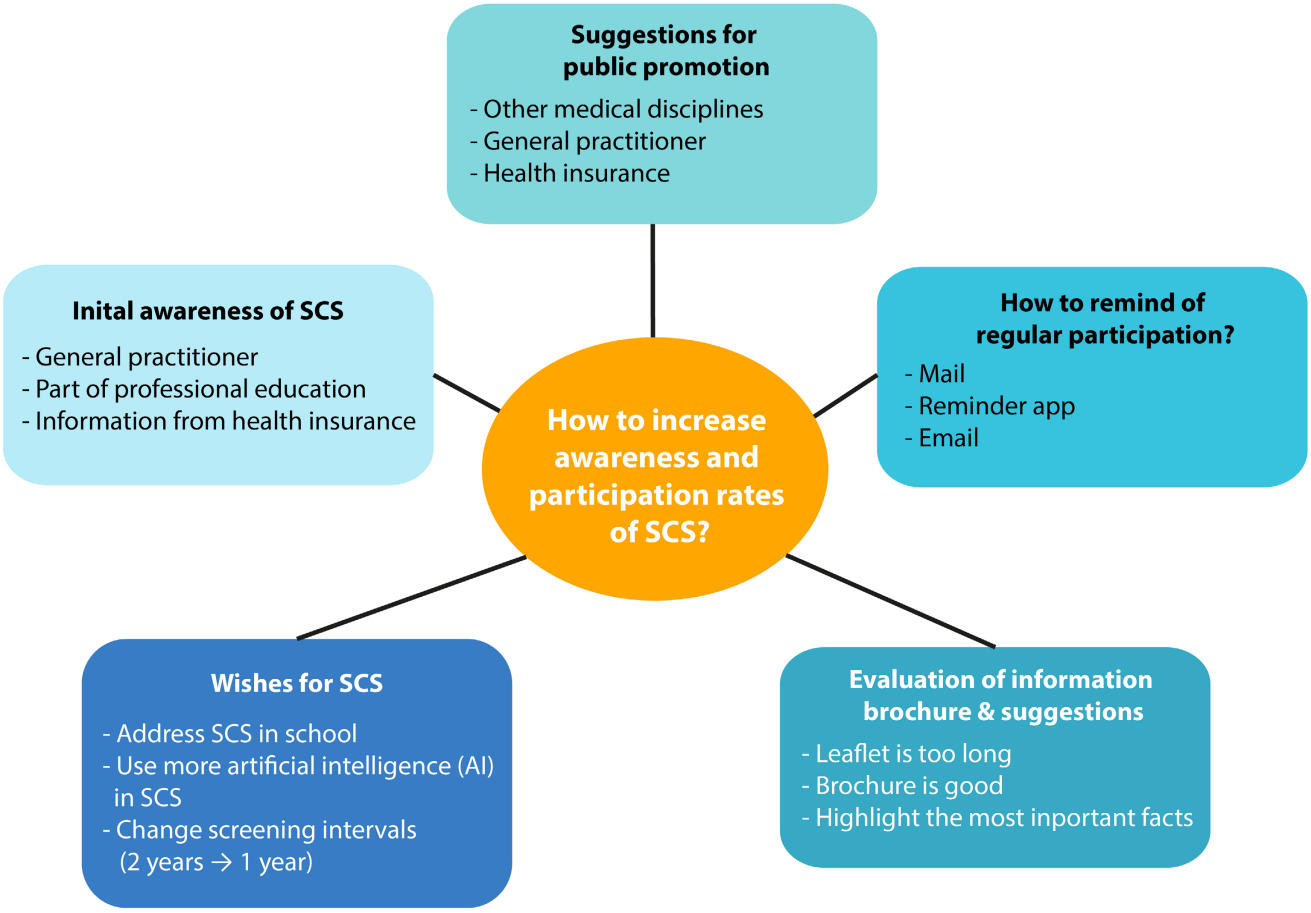
Summary of the key findings of the project.

Overall, improving information about SCS eligibility can be achieved by simple means and can remedy ignorance (Figure 1). Our qualitative approach is the first sub‐project of the HELIOS study and revealed numerous ideas from entitled persons on how to increase awareness of the possibility of SCS. In the second part of the project, a quantitative approach will be developed based on the participants' suggested opportunities to raise awareness of SCS.^20^ Further education of the population regarding skin cancer with its potentially lethal outcome is necessary. Overall, the results of this project will inform stakeholders to take appropriate actions and will contribute to increasing participation rates in SCS, promoting early detection of skin cancer.

## AUTHOR CONTRIBUTIONS

**Theresa Steeb:** Conceptualization (equal); data curation (equal); formal analysis (equal); funding acquisition (equal); investigation (equal); methodology (equal); project administration (equal); software (equal); validation (equal); visualization (equal); writing – original draft (lead). **Anja Wessely:** Data curation (equal); visualization (lead); writing – review and editing (equal). **Markus V. Heppt:** Data curation (equal); methodology (equal); project administration (equal); resources (equal); supervision (equal); writing – review and editing (equal). **Michael Erdmann:** Resources (equal); writing – review and editing (equal). **Stefanie J. Klug:** Conceptualization (equal); funding acquisition (equal); writing – review and editing (equal). **Carola Berking:** Conceptualization (equal); data curation (equal); funding acquisition (lead); project administration (equal); resources (lead); writing – review and editing (equal).

## FUNDING INFORMATION

This research was supported by a grant from the German Cancer Aid (grant number 70113659).

## CONFLICT OF INTEREST STATEMENT

The authors declare no conflict of interest. The funders had no role in the design of the study; in the collection, analyses or interpretation of data; in the writing of the manuscript, or in the decision to publish the results.

## CLINICAL TRIAL REGISTRATION

Not applicable.

## INFORMED CONSENT STATEMENT

Informed consent was obtained from all subjects involved in the study.

## INSTITUTIONAL REVIEW BOARD STATEMENT

The study was approved by the institutional review board of the University Hospital Erlangen (Vote number: 188_20B).

## Data Availability

The data that support the findings of this study are available from the corresponding author upon reasonable request.
